# *Salmonella*-derived OMVs as a vaccine platform for *Riemerella anatipestifer* outer membrane proteins to prevent infection in ducks

**DOI:** 10.1128/aem.00608-26

**Published:** 2026-06-24

**Authors:** Guodong Zhou, Zhihui Zhang, Zhili Luo, Shengliang Cao, Yubao Li

**Affiliations:** 1 College of Agriculture and Biology, Liaocheng University 58291, Liaocheng, Shandong, China; 2 Shandong Key Laboratory of Applied Technology for Protein and Peptide Drugs, Liaocheng University 58291, Liaocheng, Shandong, China; 3 College of Pharmaceutical Sciences and Food Engineering, Liaocheng University 58291, Liaocheng, Shandong, China; Centers for Disease Control and Prevention, Atlanta, Georgia, USA

**Keywords:** OMVs, Lpp, *Riemerella anatipestifer*, membrane proteins, PorQ, YaeT, YiaD

## Abstract

*Riemerella anatipestifer* (RA) is a major pathogen in waterfowl, responsible for severe septicemia and serositis, leading to significant economic losses. As antibiotic resistance becomes more widespread, there is an urgent need for effective, novel vaccine strategies. In this study, we engineered three recombinant RA antigens, PorQ, YaeT, and YiaD, onto the surface of outer membrane vesicles (OMVs) derived from a *Salmonella* Typhimurium mutant strain using the lipoprotein signal peptide (Lpp). The engineered OMVs were shown to induce robust humoral and cellular immune responses in ducks. Immunization with these OMVs resulted in 80% survival for ducks immunized with OMVs displaying YaeT and YiaD antigens and 70% survival for ducks immunized with OMVs displaying PorQ, following challenge with RA-SD-1 (serotype 2). Furthermore, ducks immunized with OMVs displaying YaeT and YiaD exhibited reduced pathological damage and lower bacterial loads in key organs. These findings demonstrate that OMVs displaying YaeT and YiaD provide strong protection against RA infections, suggesting the potential of OMVs as an effective vaccine platform for waterfowl across various RA serotypes.

**IMPORTANCE:**

The study addresses a growing challenge in poultry health, as *Riemerella anatipestifer* (RA) infections continue to cause substantial losses in waterfowl farming. The development of novel vaccines is crucial due to the rise in antibiotic resistance. This study highlights the potential of using outer membrane vesicles (OMVs) as a versatile vaccine platform to display multiple RA antigens. The promising results, including high survival rates and reduced organ damage, indicate that OMVs could provide an effective means to prevent RA infection in waterfowl, paving the way for future vaccine development targeting various serotypes of RA.

## INTRODUCTION

*Riemerella anatipestifer* (RA), a gram-negative bacterium, is a significant pathogen responsible for septicemia in waterfowl, particularly domestic ducks and geese (1). This pathogen leads to substantial economic losses in the poultry industry worldwide, causing increased mortality, reduced growth, and escalating treatment costs, especially in ducks aged 1–8 weeks. In recent years, a concerning trend of severe infections has also been observed in chickens, further exacerbating the economic impact of RA (2, 3). The pathogen’s ability to infect multiple poultry species highlights the widespread threat it poses. Moreover, the presence of *R. anatipestifer* in chickens raises concerns about potential contamination of poultry meat during slaughter and processing, posing a potential risk to food safety and public health (4). RA’s serotypic diversity, with 21 serotypes identified, complicates the development of effective control measures (5, 6). Serotypes 1 and 2 are recognized for their marked pathogenic potential and have been reported in multiple countries, including China (7). The emergence and spread of RA infections have highlighted the urgent need for novel control strategies, such as bacteriophage therapy and approaches based on understanding host antimicrobial defense mechanisms (8–10).

Numerous novel antimicrobial treatments are emerging, but bacterial vaccines continue to be regarded as one of the most reliable strategies for infection prevention and control (11–15). *Riemerella anatipestifer* exhibits pathogenicity through various virulence factors, including adhesins, toxins, and outer membrane proteins, which are major contributors to its pathogenicity in waterfowl (16). Outer membrane proteins have been identified as ideal target antigens for the development of protective vaccines against RA (16). Among them, YaeT, identified from the RA core genome, stands out due to its broad immunoreactivity and high sequence conservation, making it a promising candidate with potential cross-protective value (17). Similarly, PorQ and YiaD have been recognized as potential vaccine candidates for their ability to induce immune responses and provide protection against RA infection (18). These findings suggest that YaeT, PorQ, and YiaD are ideal target antigens for RA vaccine development. However, recombinant protein-based vaccines typically require the inclusion of specific adjuvants to generate strong protective immunity (19). Several adjuvants have been approved for use in vaccines, including mineral salts (such as aluminum salts), emulsions (e.g., MF59 and ISA 206), liposomes, microbial derivatives (such as CpG and rSly), and cytokines (such as dendritic cells and IL-12) (20, 21). The choice of adjuvant strategy, however, is dependent on the type of vaccine, as different vaccines require tailored adjuvant formulation.

Outer membrane vesicles (OMVs), naturally occurring non-replicative nanoparticles released by gram-negative bacteria, range in size from 30 to 250 nm (22). They serve as key regulators of bacterial homeostasis and communication and exhibit remarkable immunostimulatory properties due to their inherent pathogen-associated molecular patterns (PAMPs), making them potential pathogen-mimicking adjuvants (23, 24). Despite the limitations in their immunogenicity for direct drug delivery applications, OMVs offer distinct advantages as vaccine carriers, including their small size, ease of accumulation in lymph nodes, and the potential for large-scale production through bacterial cultures (23, 24). These characteristics make OMVs highly attractive candidates for vaccine development. Through genetic engineering, antigens from various pathogens can be integrated into OMVs (25–27). Additionally, modified live attenuated *Salmonella* strains have also been used as significant sources of OMVs, playing a crucial role in delivering antigens for a variety of infectious diseases (28, 29). Engineered OMVs can induce the production of pathogen-specific antibody responses, demonstrating their substantial potential as a vaccine platform that stimulates humoral immunity. These findings further highlight the promising applications of OMVs in the field of vaccine development.

A growing body of evidence suggests that immune responses triggered by extracellular antigens are more potent than those elicited by intracellular antigens (30–32). Lpp (Braun's lipoprotein), a highly abundant bacterial lipoprotein found in the outer membrane, exists in two distinct forms (33, 34). The conjugated form, which accounts for approximately one-third of the total Lpp, is located in the periplasm and covalently cross-linked with peptidoglycan, while the free form, comprising around two-thirds of the total Lpp, is inserted into the outer membrane with its C-terminal exposed on the surface (33, 34). It has been demonstrated that the free form of Lpp in *Escherichia coli* is capable of presenting the FLAG epitope on the cell surface (34). Furthermore, it has been shown that when *E. coli* expresses lipidated FhuD2 with the Lpp lipoprotein signal peptide, the protein is surface-exposed (35). In our previous work, the *Streptococcus suis* SaoA protein, containing the Lpp signal peptide, was anchored to the surface of live attenuated *Salmonella* (29). After OMVs were extracted, it was confirmed that the protein was predominantly localized to the OMV surface.

In this study, we established a vaccine platform based on engineered *Salmonella* OMVs and utilized the Lpp signal sequence (*lpp* ss) to display target antigens on the OMV surface. We confirmed that the *lpp* ss-mediated surface display of RA antigens effectively induced high levels of antibody responses and cellular immunity. Ducks immunized with these engineered OMVs exhibited significantly enhanced pathogen clearance and demonstrated strong protection against a highly virulent strain of RA serotype 2. Leveraging this *lpp* fusion protein-based OMV surface display system, we aim to provide a more efficient method for antigen presentation and delivery, laying a foundation for the further prevention and control of RA infections.

## RESULTS

### Isolation and characterization of OMVs displaying RA antigens from engineered *Salmonella* strains

**Fig 1:**
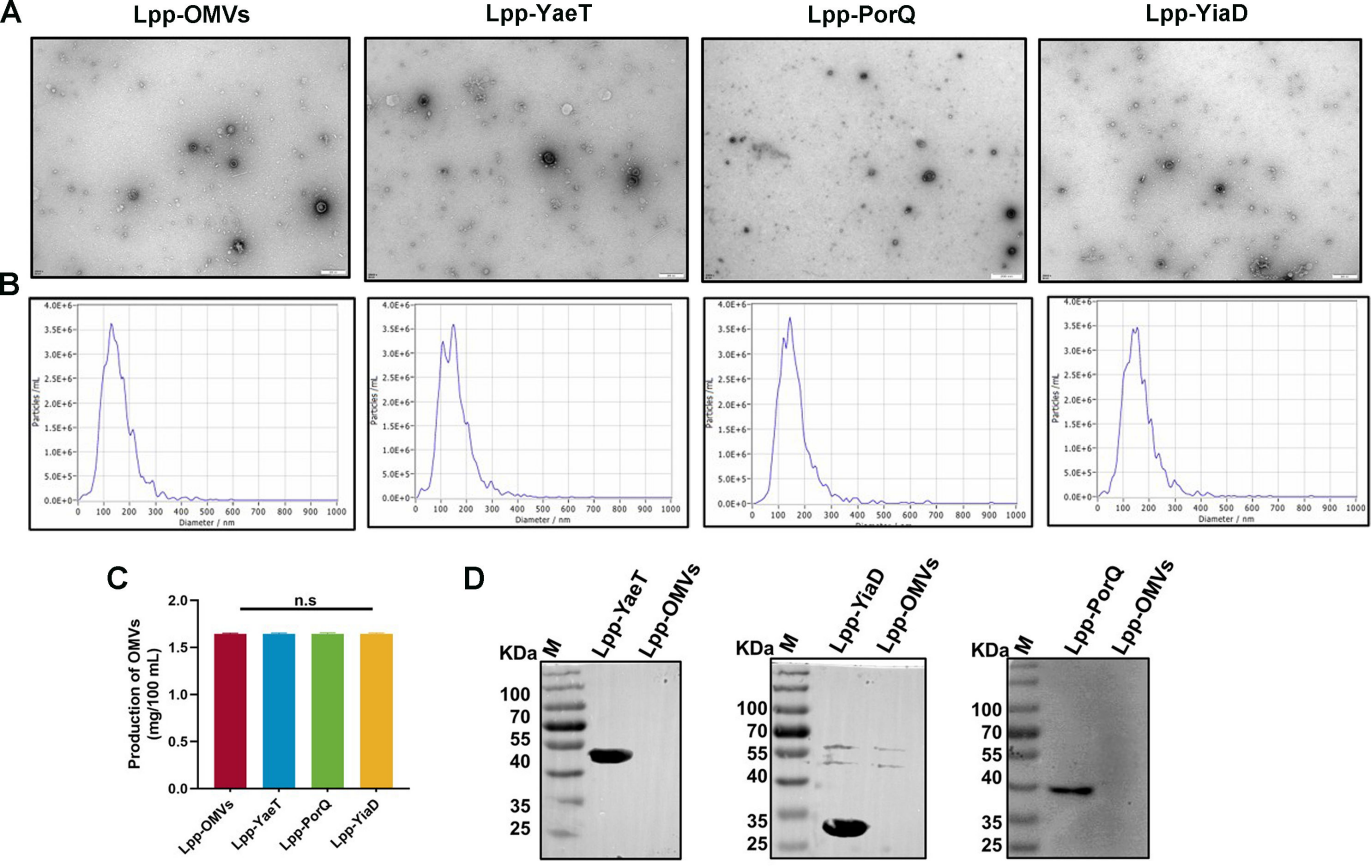
Characterization of OMVs derived from engineered *Salmonella* strains. (**A**) Transmission electron microscopy (TEM) images showing the morphology of OMVs, including those displaying recombinant antigen proteins (YaeT, PorQ, and YiaD) and OMVs without antigen proteins (Lpp-OMVs). Scale bars are indicated. (**B**) Nanoparticle tracking analysis (NTA) of OMVs from different groups, showing particle size distribution and concentration. (**C**) Quantification of OMV yield from each group. (**D**) WB analysis confirming the presence of recombinant antigen proteins (YaeT, PorQ, and YiaD) in OMVs. The results are expressed as the mean ± SD. Degrees of significance are indicated as follows: *P  <  0.05; **P  <  0.01; n.s, nonsignificant (*P* ≥ 0.05).

The engineered *Salmonella* strain RAST0013 (Δ*rfbP*Δ*fliC*Δ*fljB*Δ*ompA*) was constructed as an enhanced OMV platform for antigen delivery, with the *Riemerella anatipestifer* antigen genes *yaeT*, porQ, and *yiaD* expressed using the *lpp* signal peptide-containing pBAD33-*lpp* plasmid (data not shown). Following induction with 0.2% arabinose, OMVs were successfully extracted from RAST0013. TEM analysis revealed that these OMVs exhibited a typical bilayer structure and spherical morphology (Fig. 1A). Size distribution analysis showed that the OMVs mainly ranged from 130 to 150 nm (Fig. 1B). Quantification using the BCA protein assay further demonstrated that incorporation of antigen proteins did not significantly affect OMV production, with both antigen-loaded and control OMVs yielding approximately 1.64 ± 0.1242 mg per 100 mL of culture (Fig. 1C).

Western blot analysis confirmed the expression of all three recombinant antigens (YaeT, PorQ, and YiaD) in the OMVs, with the expected band sizes of ~40 kDa for YaeT, ~35 kDa for PorQ, and ~25 kDa for YiaD (Fig. 1D). In contrast, OMVs extracted from the control strain, which did not express the antigen genes, showed no detectable antigen expression, as shown in Fig. 1D, consistent with the expected results.

### Safety assessment of OMVs displaying antigen proteins shows low cytotoxicity and no impact on duck growth performance

**Fig 2:**
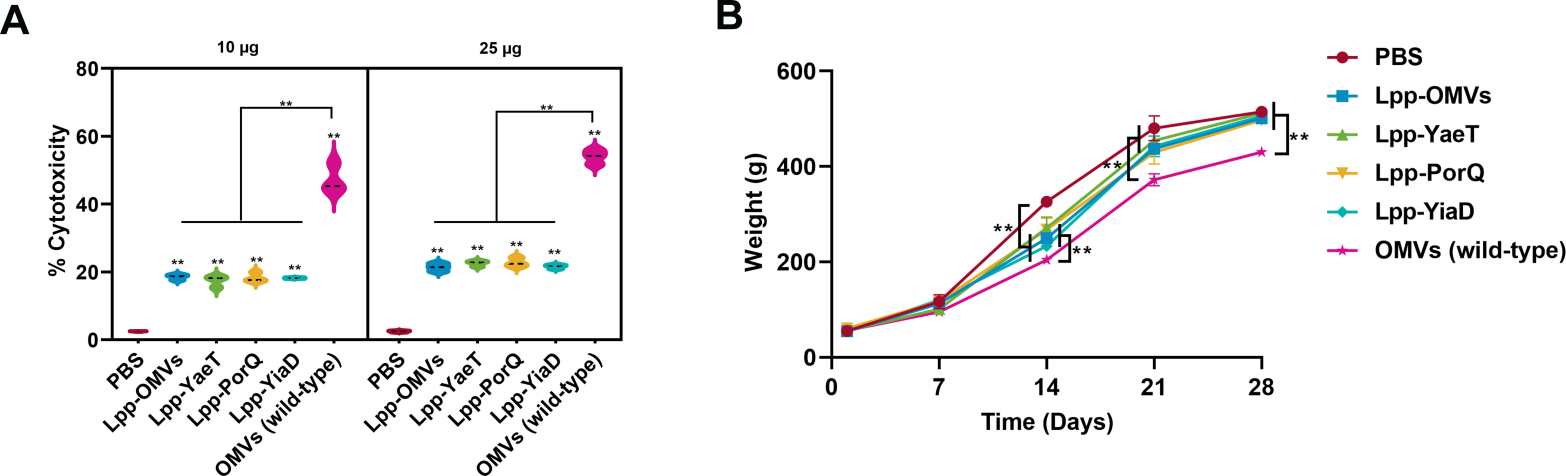
Evaluation of the safety profile of OMVs *in vitro* and *in vivo*. (**A**) LDH release assay in RAW264.7 cells treated with 10 or 25 µg of OMVs. OMVs (wild-type) were isolated from the parental *S*. Typhimurium ATCC14028 strain, while Lpp-OMVs and antigen-displaying OMVs were isolated from engineered *Salmonella* strains. LDH levels in the culture supernatants were measured to assess cytotoxicity. (**B**) Body weight monitoring of ducklings after immunization with 100 µg of OMVs (antigen-free or displaying YaeT, PorQ, or YiaD). The results are expressed as the mean ± SD.Degrees of significance are indicated as follows: **P < 0.01; n.s, nonsignificant (*P* ≥ 0.05).

Lactate dehydrogenase (LDH) is a stable enzyme located in the cytoplasm of all normal cells. When cells are damaged and their membrane integrity is compromised, LDH is released into the culture medium (36, 37). The amount of LDH in the supernatant can serve as an indicator of cell membrane damage and cytotoxicity. To assess the potential cytotoxic effects of OMVs, we measured the release of LDH from RAW264.7 cells in the presence of OMVs, either carrying no antigen or displaying one of the three recombinant antigens (YaeT, PorQ, or YiaD). OMVs were tested at concentrations of 10 and 25 µg. The results, as shown in Fig. 2A, demonstrated no significant increase in LDH release at either concentration compared with the untreated control group, indicating that OMVs, regardless of antigen display, did not induce significant cellular damage.

In addition to the LDH assay, we also assessed the effect of OMV immunization on the body weight gain of ducklings. Ducklings were immunized with 100 µg of OMVs, either displaying YaeT, PorQ, or YiaD antigens, or without antigens. No significant differences in body weight gain were observed between the immunized groups and the control group throughout the experimental period (Fig. 2B). These results suggest that OMV vaccination, regardless of antigen presence, did not adversely affect the growth or weight gain of the ducklings.

### Evaluation of antibody titers in ducks immunized with OMVs displaying recombinant antigens

**Fig 3:**
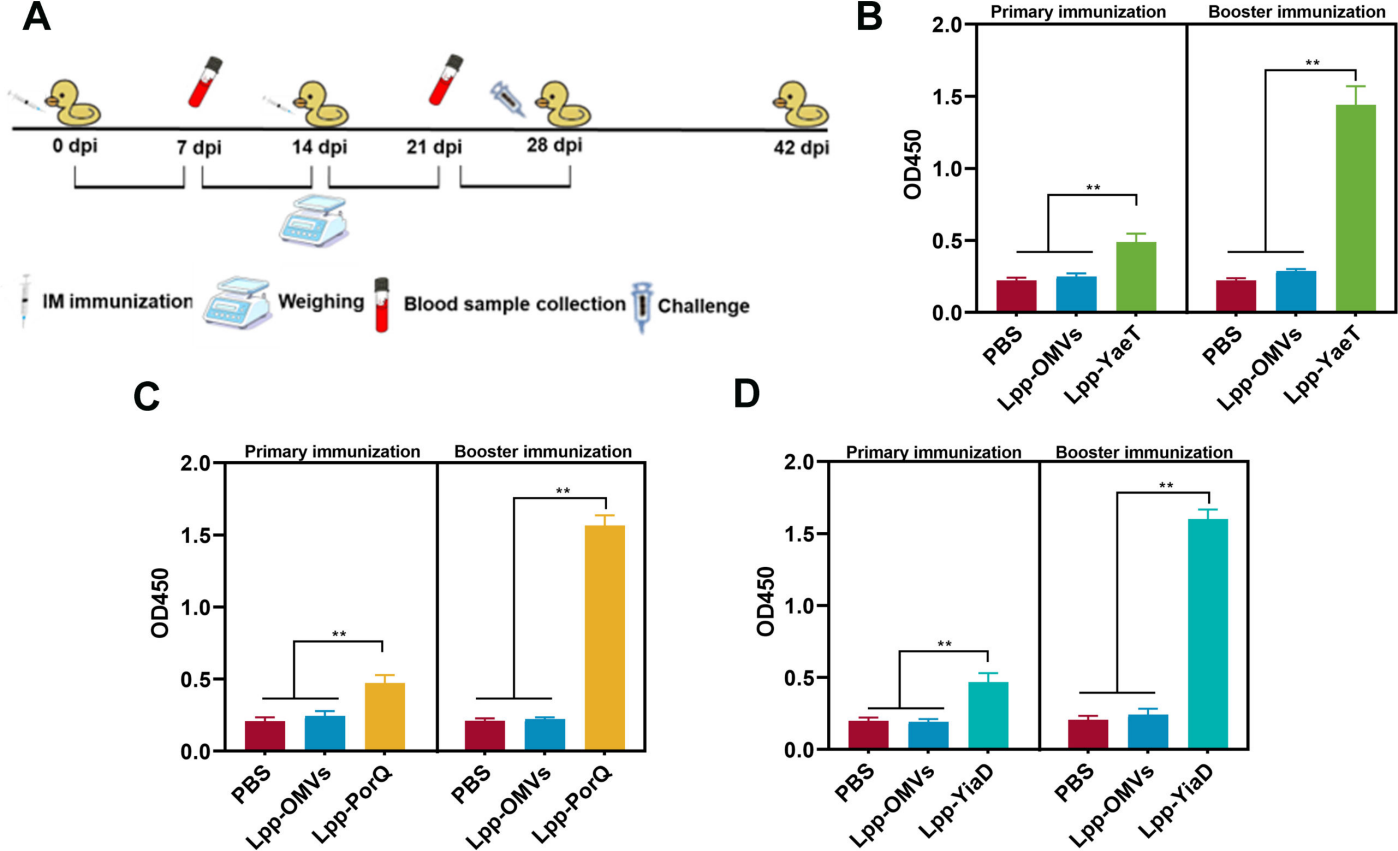
Humoral immune responses induced by OMVs displaying recombinant antigens. (**A**) Schematic illustration of the immunization and blood collection schedule. (**B–D**) Antigen-specific IgY antibody responses measured by indirect ELISA in sera collected from ducks immunized with OMVs without antigen proteins, OMVs displaying YaeT, PorQ, or YiaD, and a PBS control group. Antibody titers against YaeT (**B**), PorQ (**C**), and YiaD (**D**) were determined at 7 days after primary immunization and 7 days after booster immunization (days 7 and 21). The results are expressed as the mean ± SD.Degrees of significance are indicated as follows: **P < 0.01; n.s, nonsignificant (*P* ≥ 0.05).

Sera collected from ducks in five groups, including those immunized with OMVs without antigen proteins, OMVs displaying YaeT, PorQ, or YiaD antigens, and a PBS control group, were analyzed by indirect ELISA to determine antigen-specific IgY responses. The immunization and blood collection schedule is shown in Fig. 3A. As shown in Fig. 3B through D, antibody titers were significantly higher in the OMV groups displaying YaeT, PorQ, and YiaD compared to the blank control group at both 7 days post-primary immunization and 7 days following booster immunization (days 7 and 21). No detectable antibody response was observed in the blank control group at either time point.

### Enhancement of lymphocyte proliferation and cytokine production by OMVs displaying antigen proteins

**Fig 4:**
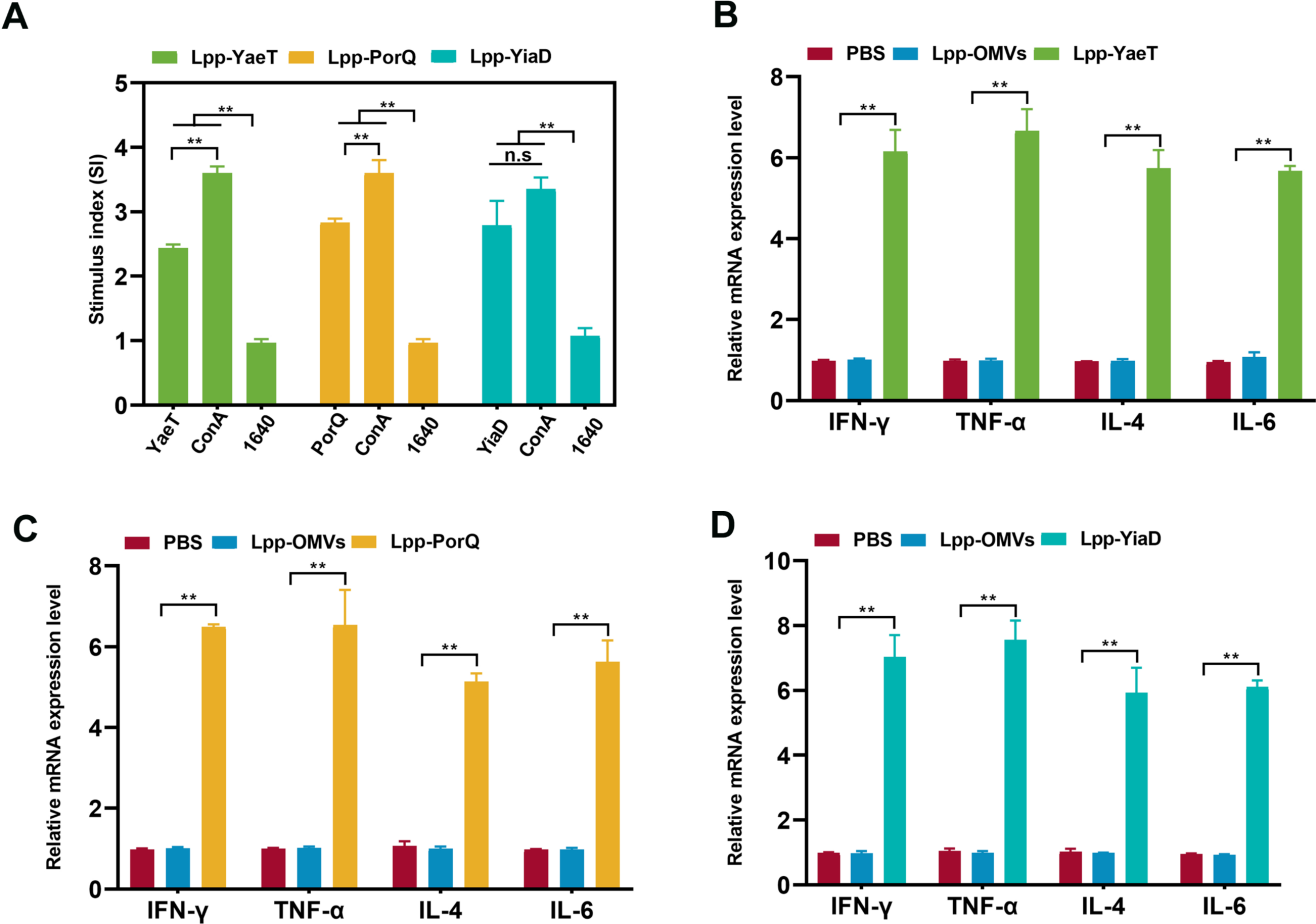
Cellular immune responses induced by OMV-based vaccination. (**A**) Lymphocyte proliferation assay of PBMCs isolated from ducks in different groups. PBMCs were stimulated *in vitro* with the corresponding recombinant antigens for 24 h, and cell proliferation was measured using a CCK-8 assay. Stimulation indices (SIs) were calculated. (**B–D**) Relative mRNA expression levels of cytokines in PBMCs stimulated with different recombinant antigens, as determined by qRT-PCR. Cytokine expression profiles following stimulation with YaeT (**B**), PorQ (**C**), and YiaD (**D**) were analyzed, including Th1-type cytokines (IFN-γ and IFN-α) and Th2-type cytokines (IL-4 and IL-6). The results are expressed as the mean ± SD.Degrees of significance are indicated as follows: **P < 0.01; n.s, nonsignificant (*P* ≥ 0.05).

Seven days after the booster immunization, PBMCs were isolated from ducks in all five groups those immunized with OMVs displaying YaeT, PorQ, and YiaD antigens, OMVs without antigen proteins, and a PBS group. A single-cell suspension was prepared for each group. The PBMCs were then stimulated *in vitro* with the corresponding recombinant antigen proteins for 24 h in the presence of 5% CO_2_ at 37°C. Lymphocyte proliferation was measured using a CCK-8 assay, and stimulation indices (SIs) were calculated. PBMCs from ducklings immunized with OMVs carrying YaeT, PorQ, or YiaD exhibited strong lymphocyte proliferation after stimulation with the corresponding recombinant antigens (Fig. 4A). In contrast, no significant lymphocyte proliferation was observed in the PBS or Lpp-OMVs groups following stimulation with the same antigens (Fig. S1). These results indicate that OMVs carrying the different RA antigens induced robust antigen-specific cellular immune responses.

To further evaluate the cellular immune response, the expression of Th1-type cytokines (IFN-γ and IFN-α) and Th2-type cytokines (IL-4 and IL-6) was measured by qRT-PCR following *in vitro* antigen stimulation. As shown in Fig. 4B through D, all antigen-stimulated groups exhibited elevated levels of these cytokines, demonstrating that vaccination effectively induced robust helper T cell responses (Th1 and Th2).

### OMVs immunization significantly reduces bacterial load in blood and liver of ducks post-RA challenge

**Fig 5:**
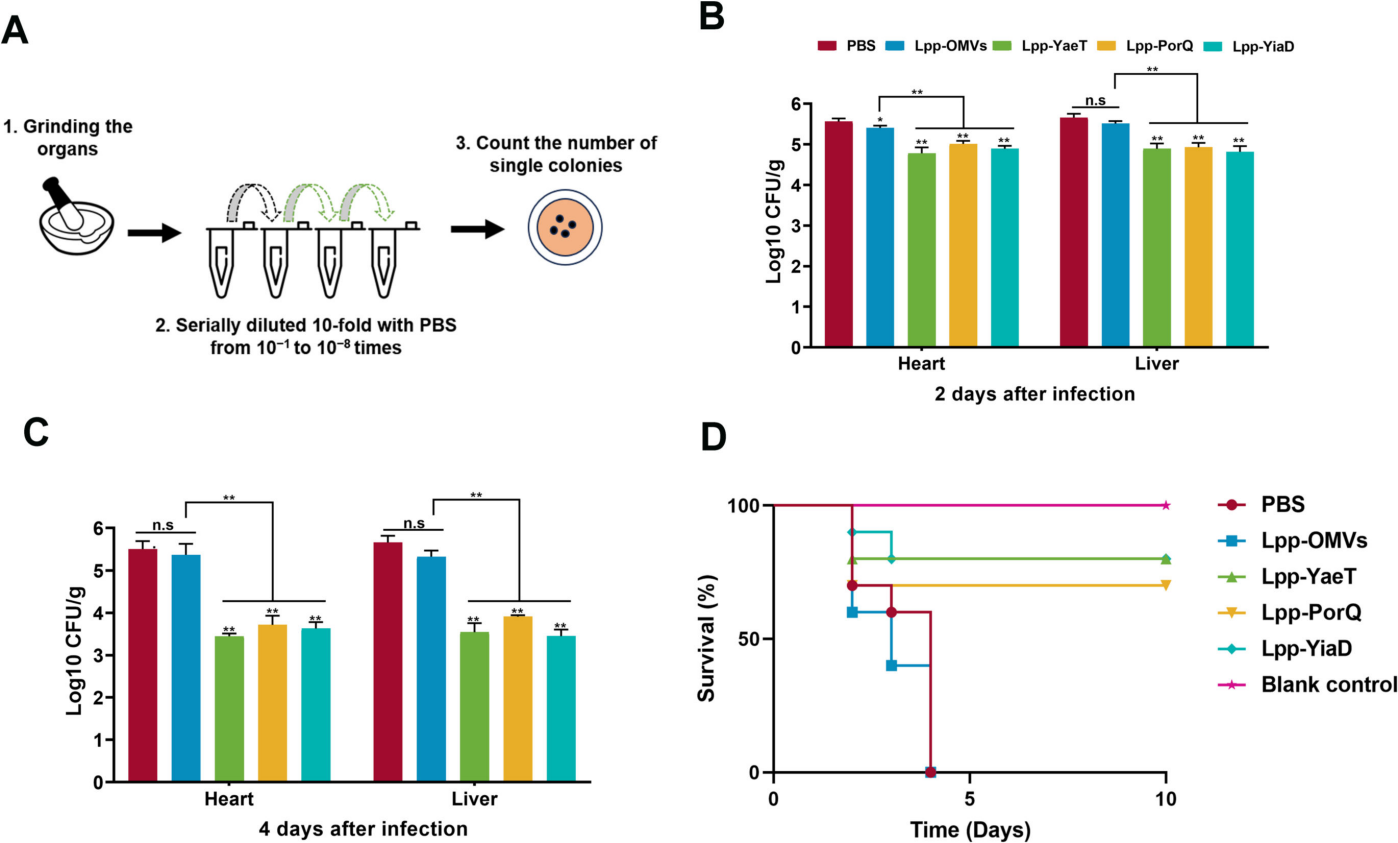
Protective efficacy of OMVs displaying recombinant antigens against RA infection. (**A**) Schematic illustration of bacterial load determination in duck organs. Heart and liver tissues were collected, homogenized, serially diluted, and plated for colony-forming unit (CFU) enumeration. (**B**) Bacterial loads in the heart and liver of ducklings at 2 days post-challenge with RA strain RA-SD-1. (**C**) Bacterial loads in the heart and liver of ducklings at 4 days post-challenge. (**D**) Survival curves of ducklings following challenge with RA-SD-1 over a 14-day observation period. The results are expressed as the mean ± SD. Degrees of significance are indicated as follows: *P < 0.05; **P < 0.01; n.s, nonsignificant (*P* ≥ 0.05).

Bacterial colonization in the heart and liver of ducklings immunized with OMVs displaying YaeT, YiaD, or PorQ was assessed at 2 and 4 days post-challenge (Fig. 5A). Notably, all three antigen groups exhibited a significant reduction in bacterial loads compared to the PBS control (Fig. 5B and C). A moderate decrease was already evident at day 2, whereas by day 4, bacterial burdens in both organs were further diminished, indicating efficient bacterial clearance. In contrast, ducklings receiving Lpp-OMVs without antigens displayed markedly higher bacterial loads than the antigen-containing groups at both time points, reflecting limited protective efficacy in the absence of antigenic stimulation.

### Protection against RA infection induced by recombinant proteins

To evaluate the protective efficacy of OMVs displaying recombinant antigen proteins, ducks were immunized with OMVs carrying YaeT, YiaD, or PorQ antigens. Two control groups were included: one group received OMVs without antigen proteins (Lpp-OMVs) as a vector control, and another group was immunized with PBS and served as the challenge control. Following a challenge with RA-SD-1, the survival rates of immunized ducks were monitored over a 14-day period. In the group immunized with OMVs displaying YaeT and YiaD, 80% of the ducks survived the full observation period (Fig. 5D). The group immunized with OMVs displaying PorQ showed a survival rate of 70%, while the control group had a survival rate of 0% (Fig. 5D). These results emphasize the promising protective effects of OMVs displaying YaeT and YiaD, making them strong candidates for future vaccine development.

### OMVs displaying YaeT antigen provide effective protection against organ damage and pathological changes induced by RA infection

**Fig 6:**
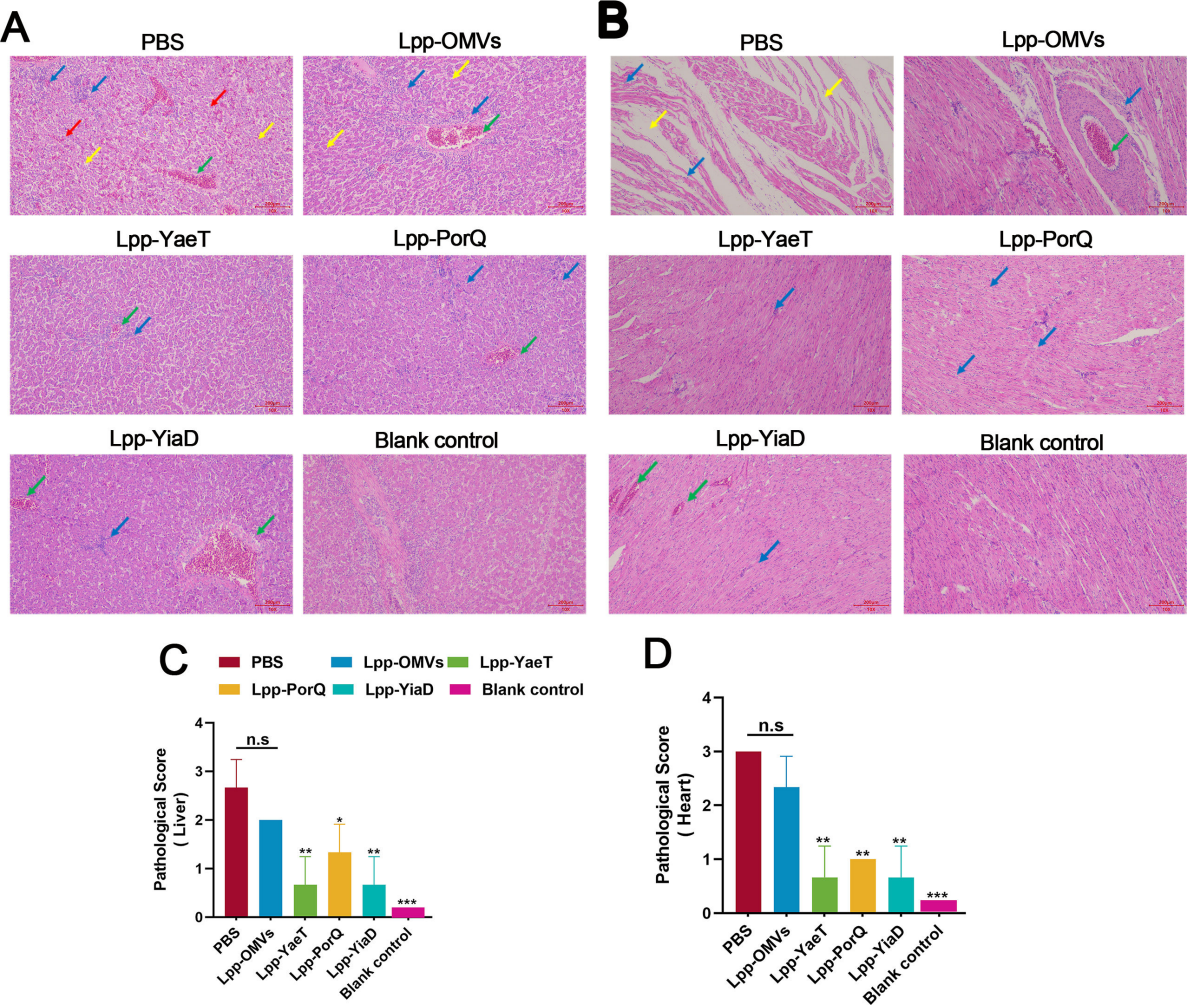
Histopathological evaluation of heart and liver tissues following RA challenge. (**A**) Representative gross and histopathological images of liver tissues from different groups. Severe pathological lesions were observed in the PBS control group, including hepatocyte edema (yellow arrows), sinusoidal congestion (red arrows), inflammatory cell infiltration (blue arrows), and vascular congestion (green arrows). (**B**) Representative gross and histopathological images of heart tissues from different groups. Yellow arrows indicate loose arrangement of myocardial fibers, blue arrows indicate lymphocyte infiltration, and green arrows indicate congestion. (**C**) Histopathological scoring of liver tissues across different groups. (**D**) Histopathological scoring of heart tissues across different groups. The results are expressed as the mean ± SD. Degrees of significance are indicated as follows: *P < 0.05; **P < 0.01; n.s, nonsignificant (*P* ≥ 0.05).

Pathological changes in the heart and liver of immunized ducks were assessed following challenge with RA-SD-1. Upon dissection, significant anatomical changes were observed in the control group, which had no antigen exposure. Both the liver surface and epicardium exhibited yellow fibrinous exudate, indicating substantial tissue damage (Fig. 6A and B). Histopathological analysis confirmed extensive damage. In the liver, hepatocytes showed cytoplasmic dissolution, necrosis, unclear cell boundaries, and interstitial hemorrhage, while the heart displayed pronounced myocardial fiber degeneration, inflammatory cell infiltration, and hemorrhagic foci. The epicardium also exhibited yellow fibrinous exudate, further indicating severe inflammation and tissue destruction.

In contrast, immunized groups exhibited significantly reduced pathological alterations (Fig. 6A and B). Ducks immunized with OMVs displaying YaeT showed the least damage, with no yellow fibrinous exudate on the liver surface or epicardium. Microscopic examination revealed well-preserved hepatocyte structures, mild vacuolar degeneration, and minimal inflammatory cell infiltration in the liver. The heart showed minimal myocardial fiber infiltration and slight hemorrhaging, accompanied by fewer inflammatory cells compared to the control group. Ducks immunized with OMVs displaying YiaD and PorQ also exhibited reduced pathology, but their protection was less pronounced than that of the YaeT group. In these groups, the liver showed mild swelling, cellular necrosis, and minor inflammatory infiltration, while the heart exhibited mild myocardial fiber degeneration and slight hemorrhages.

The YaeT-immunized group exhibited the lowest pathology scores for both liver and heart tissues, indicating the most effective protection against RA infection (Fig. 6C and D). These findings highlight the superior protective efficacy of OMVs displaying YaeT, positioning it as a promising candidate for future vaccine development.

## DISCUSSION

*Riemerella anatipestifer* infections pose a significant threat to the global poultry industry, particularly in duck farming, leading to substantial economic losses (1, 16). Characterized by infectious serositis, the disease results in high mortality rates and reduced productivity (16). While traditionally associated with ducks, recent studies have shown that RA can also infect chickens, further broadening its impact on poultry farming (2, 38). Despite the use of antibiotics to control these infections, the rising issue of antimicrobial resistance has compromised treatment efficacy, particularly in regions with limited access to effective drugs (8, 39, 40). Vaccination remains the most effective strategy for managing RA; however, current vaccines offer limited protection, especially against diverse serotypes (41–43). The ability of RA to infect multiple poultry species, coupled with the emergence of antibiotic resistance, highlights the need for more effective, broad-spectrum vaccines. Protein-based vaccines, particularly recombinant protein approaches, have shown promising potential in improving disease control and reducing reliance on antibiotics (44). However, the specific recombinant protein that offers the most effective protection remains unclear, and further research is needed to identify the optimal candidates for vaccine development (8, 18).

OMVs have emerged as promising vaccine platforms because they naturally contain multiple pathogen-associated molecular patterns, including OMPs, lipopolysaccharides, and periplasmic proteins, which can effectively stimulate both innate and adaptive immune responses (26, 45). Their bilayer lipid structure protects antigenic proteins from rapid degradation and facilitates efficient antigen delivery to immune cells, while also promoting the maturation of antigen-presenting cells such as dendritic cells, which are essential for initiating both naïve and memory immune responses (46). Engineering bacterial strains to optimize OMV production and antigen presentation has therefore become an important strategy for improving OMV-based vaccines (26, 45, 47). In this study, OMVs were derived from an engineered *Salmonella* strain carrying deletions of *rfbP*, *fliC*, *fljB*, and *ompA*. Previous studies have shown that deletion of *rfbP* remodels the outer membrane structure and enhances OMV production, thereby increasing vesicle availability for antigen delivery (28). Meanwhile, deletion of *fliC* and *fljB* effectively reduces flagellin contamination in OMVs, preventing excessive immune responses against flagellar proteins and allowing the immune system to focus more effectively on the target antigens (48). In addition, deletion of *ompA* has been reported to promote OMV release and alter membrane stability, further facilitating vesicle formation (49, 50). Together, these genetic modifications provide an optimized OMV platform for efficient antigen presentation and vaccine delivery.

OMPs are key vaccine targets due to their strong immunogenicity and accessibility to the host immune system, and their surface localization enhances recognition by immune cells while contributing to bacterial virulence (51–53). The magnitude and quality of the immune response depend not only on the intrinsic properties of the antigen but also on its subcellular localization (30–32). Previous studies have shown that surface-exposed antigens elicit stronger humoral and cellular responses, and our prior work demonstrated that anchoring SaoA from *Streptococcus suis* toward the bacterial surface using an N-terminal Lpp signal sequence led to higher antigen-specific antibody levels and more robust immune activation (29, 31). In this study, YaeT, YiaD, and PorQ were selected as candidate antigens because they are membrane proteins of RA and were fused with an N-terminal Lpp lipoprotein signal sequence for expression in the engineered *Salmonella* strain, thereby promoting their anchoring toward the OMV surface and facilitating efficient antigen presentation. While Lpp-mediated lipidation does not guarantee complete surface exposure, presentation within the OMV membrane likely provides partial accessibility, facilitating immune recognition. Flow cytometry analyses from previous studies support that a fraction of lipidated antigens can be surface-accessible, and the high antibody and cytokine responses observed in this study suggest functional recognition by immune cells (29, 35). Further studies are warranted to confirm the precise topology and extent of surface exposure of these engineered antigens.

Consistent with the enhanced immune responses, vaccination with YaeT-OMVs conferred the highest level of protection in challenged ducks, as reflected by improved survival rates and markedly reduced clinical symptoms and pathological lesions. The superior protective efficacy of YaeT may be attributed to its strong antigenicity and the presence of prominent B-cell epitopes, which facilitate the generation of high-affinity antibodies. These antibodies may enhance complement activation and promote phagocytic clearance of RA, thereby contributing to effective immune defense (17). Together with the observed induction of helper T-cell responses (Th1 and Th2), these findings highlight YaeT as a promising immunogen and further support the potential of engineered OMVs as an effective vaccine delivery platform against RA.

Nevertheless, several aspects of this study warrant further investigation. Although three outer membrane proteins were evaluated as candidate antigens, additional immunogenic proteins from RA should be explored in future studies to further improve vaccine efficacy and broaden cross-serotype protection. Moreover, the antigen presentation strategy within the OMV platform could be further optimized to enhance antigen exposure and immune stimulation. In the present study, the protective efficacy of the vaccine was evaluated using a single RA strain in a duck challenge model. Considering the genetic diversity among different serotypes of RA, future studies should assess the cross-protective capacity of YaeT-OMVs against multiple epidemiologically relevant strains. In addition, more detailed immunological analyses, including long-term immune memory evaluation and comprehensive pathological assessments, will be necessary to better understand the durability, safety, and protective mechanisms of the vaccine.

In conclusion, our study demonstrates that engineered OMVs derived from a mutant *Salmonella* strain can serve as an effective antigen delivery platform for RA vaccine development. Among the three evaluated outer membrane proteins, YaeT induced the strongest immune responses and conferred the highest level of protection against experimental challenge in ducks. These findings highlight YaeT as a promising vaccine antigen and support the potential of OMV-based strategies for controlling RA infections. Furthermore, the engineered OMV platform described here may provide a versatile delivery system for presenting antigens from other poultry pathogens, offering a promising direction for the development of next-generation multivalent vaccines in the poultry industry.

## MATERIALS AND METHODS

### Bacterial cultivation, recombinant protein production, and vaccine formulation

The bacterial strains and plasmids used in this study are listed in Table 1. The Serotype 2 RA strain (RA-SD-1), previously isolated from diseased ducks and maintained in our laboratory, was used as the challenge strain in subsequent protection assays (54). Furthermore, DNA was extracted from the RA-SD-1 and used as the template for amplifying the genes encoding the antigen proteins. The strain was cultured in tryptic soy broth (TSB; Difco, Detroit, MI, USA) or on tryptic soy agar (TSA) plates supplemented with 5% calf serum (Gibco, USA) at 37 °C for 18–24 h under anaerobic conditions using a commercial anaerobic gas-generation system. The *Salmonella* Typhimurium strain ATCC14028 was first genetically modified and subsequently used for OMV isolation. The donor strain *E. coli* χ7213 and the suicide vector plasmid pRE112 used for allelic exchange were obtained from Baosai Biotechnology (China). The pBAD33-*lpp* plasmid, which contains the Lpp signal sequence and a His tag, was synthesized by a biotechnology company and is stored in our laboratory. This plasmid was subsequently used for the cloning and expression of the YaeT, PorQ, and YiaD proteins. When required, the culture medium was supplemented with 50 μg/mL chloramphenicol (Cm), and arabinose was added to a final concentration of 0.2% to induce protein expression.

**TABLE 1:** Bacterial strains and plasmids used in this study^*a*^

Strain or plasmid	Characteristics	Source or reference
*E. coli* BL21	For protein expression	Sangon Biotech (Shanghai)
*S.* Typhimurium		
ATCC14028	Wild type; virulent	Lab stock
RAST0010	Derivative strain of ATCC14028 with a deletion of the *rfbP* gene	This study
RAST0011	Derivative strain of ATCC14028 in which both the *rfbP* and *fliC* genes have been deleted	This study
RAST0012	Derivative strain of ATCC14028 in which the *rfbP*, *fliC*, and *fljB* genes have been deleted	This study
RAST0013	Derivative strain of ATCC14028 in which the *rfbP*, *fliC*, *fljB*, and *ompA* genes have been deleted	This study
*Riemerella anatipestifer* RA-SD-1	Wild type; virulent; DNA extracted for amplifying *yaeT*, *porQ*, and *yiaD* genes	(54)
Plasmids		
pBAD33-*lpp*	Modified pBAD33 plasmid that includes the lpp signal peptide for targeting the expressed protein to the bacterial surface; Cm^r^	This study
pBAD33-*lpp*- YaeT	pBAD33-*lpp* with *yaeT*; Cm^r^	This study
pBAD33-*lpp*- PorQ	pBAD33-*lpp* with *porQ*; Cm^r^	This study
pBAD33-*lpp*-YiaD	pBAD33-*lpp* with *yiaD*; Cm^r^	This study

^*a*^ Cm^r^, chloramphenicol resistance.

### Molecular and genetic procedures

Genetic modification of the ATCC14028 strain, involving the knockout of *rfbP*, *fliC*, *fljB*, and *ompA* genes, was performed to improve the OMV-induced immune response, as described in prior work (32). Suicide vectors and primers used in this study are listed in Tables 1 and 2, respectively. The genetic manipulation procedures used to generate unmarked gene deletions in ATCC14028 followed previously published methods, with minor modifications (29, 55). Briefly, SacB-based sucrose counter-selectable suicide vectors were employed to construct deletion mutants. For each deletion construct, two homologous arms flanking the target gene were amplified and purified. The resulting fragments were fused by overlap extension PCR and subsequently ligated into the pRE112 suicide plasmid. Conjugative transfer of the recombinant plasmids into *S*. Typhimurium was carried out using the donor strain χ7213. Transconjugants were selected on LB agar supplemented with chloramphenicol and lacking DAP. To obtain the second homologous recombination event, enabling excision of the integrated suicide vector, colonies were plated onto LB agar containing 5% sucrose without sodium chloride and incubated at 25°C. Successful deletion mutants were identified by colony PCR and further confirmed by Sanger sequencing.

**TABLE 2:** Primer information^*a*^

Primer name	Sequence (5′–3′)	Reference
*rfbP*-1	aagcttcttctagaggtaccCACCCATTCTGACAGATTACAG	This study
*rfbP*-2	ttcagtacttctcggtaagcTTAATCCTCACCCTCTGACC	This study
*rfbP*-3	GCTTACCGAGAAGTACT	This study
*rfbP*-4	atgaattcccgggagagctcGGATTCAACAAACTCTTTC	This study
*rfbP*-F/R	GGATCTTGTAAATACAACGCTTG/CGGTCATGGCAGGTTAAAG	This study
*fliC*-1	aagcttcttctagaggtaccCGTTCTTTGTCAGGTCTG	This study
*fliC*2	tcggtgaatcaatcgccggaGACTTGTGCCATGATCTTTTC	This study
*fliC*-3	TCCGGCGATTGATTCAC	This study
*fliC*-4	atgaattcccgggagagctcCTTAATGCCTTTGCGCCT	This study
*fliC*-F/R	CGAAATTCAGGTGCCGATAC/GTGATTTCTTTCATTACACAG	This study
*fljB*-1	aagcttcttctagaggtaccGCTACTGGTATCAATACTATC	This study
*fljB*-2	gctgaataaaacgaaataaaAAAATTTTCCTTTTGGAAGG	This study
*fljB*-3	TTTATTTCGTTTTATTCAGCC	This study
*fljB*-4	atgaattcccgggagagctcCCTGATAATTCTTCAAGAATTG	This study
*fljB*-F/R	CCCAATAGTAGCCATGATTTTCT/GTAGGAAATATCATTTAC	This study
*ompA*-1	aagcttcttctagaggtaccCGAACGTATCTCCTTTGAGTG	This study
*ompA*-2	gttttttatcagacggaaacTTTTTGCGCCTCGTTATCA	This study
*ompA*-3	GTTTCCGTCTGATAAAAAAC	This study
*ompA*-4	atgaattcccgggagagctcTGCTTAATCGGATGAAAC	This study
*ompA*-F/R	CGTTGTAGACTTTACATCGCC/CAACTGATAGAAGACGCGG	This study

^*a*^ Lowercase letters denote homologous sequences designed for overlap PCR and recombination with the plasmid backbone.

### Plasmid construction, OMVs extraction, and nanoparticle tracking analysis

The genes encoding the YaeT, PorQ, and YiaD proteins from *Riemerella anatipestifer* were amplified by PCR and directly ligated into the pBAD33-*lpp* expression vector using the Seamless Cloning Kit (C116; Vazyme). The recombinant plasmids were then transformed into a genetically engineered *Salmonella* strain for protein expression. To extract the outer membrane vesicles (OMVs), 1 mL of overnight culture was inoculated into 100 mL of LB broth supplemented with 50 μg/mL chloramphenicol and grown at 37°C to an OD_600_ of 0.5. Protein expression was induced by adding 0.2% arabinose for 24 h. After induction, the bacterial culture was harvested by centrifugation at room temperature, and the supernatant was filtered through a 0.45 μm sterile filter (Millipore, USA) to remove bacterial cells. OMVs were then collected by ultracentrifugation at 150,000 × *g* for 3 h at 4°C and washed once with sterile PBS (pH 7.4). For further purification, the OMVs were subjected to density gradient centrifugation at 150,000 × *g* for 12 h at 4°C using a discontinuous OptiPrep density gradient (20–45%). The OMV pellet was collected, resuspended in sterile PBS, and filtered again through a 0.45 μm sterile filter. To check for potential bacterial contamination, the OMVs were plated on LB agar. The quantity and quality of the OMVs were assessed by transmission electron microscopy (TEM) and quantified using a BCA protein assay kit. The size distribution and concentration of OMVs were determined by nanoparticle tracking analysis (NTA).

### SDS-PAGE and Western blot

Protein samples were separated by electrophoresis on 10% polyacrylamide gels containing sodium dodecyl sulfate (SDS), then transferred onto a PVDF membrane. The membrane was blocked with 10% non-fat milk and incubated with anti-His tag antibody at room temperature for 2 h. After washing three times with Tris-buffered saline containing 0.5% Tween 20 (Solarbio, China), the membrane was incubated with HRP-conjugated goat anti-mouse IgG at room temperature for 1 h. The immune-reactive proteins were visualized using SuperSignal West Pico chemiluminescent substrate (Thermo Scientific, Rockford, USA).

### Cytotoxicity assays

Cytotoxicity was evaluated by co-incubating the mouse macrophage cell line RAW264.7 with different concentrations of OMVs for 24 h. The cells were cultured at 37°C in 5% CO_2_ in medium containing 10% fetal bovine serum (FBS; Gibco) and supplemented with 100 μg/mL streptomycin and 100 μg/mL penicillin. RAW264.7 cells were seeded into 24-well plates and incubated for 12 h in DMEM supplemented with 10% FBS. The cells were then stimulated with 10 or 25 μg of OMVs isolated from engineered *Salmonella* strains with or without antigen expression, with OMVs derived from the wild-type *S*. Typhimurium strain ATCC14028 and PBS used as controls, and incubated overnight. PBS was used as a negative control. Following the manufacturer’s protocol, supernatants were collected and analyzed for lactate dehydrogenase (LDH) release using a cytotoxicity detection kit (Sigma). Maximum LDH release was achieved by lysing the cells with 1% Triton X-100, while spontaneous release from uninfected cells was used as a baseline control. Cytotoxicity was calculated using the following formula: % cytotoxicity = [(experimental release − spontaneous release)/(maximum release − spontaneous release)] × 100%.

### Immunization and challenge protocol

One-day-old Cherry Valley ducklings were randomly assigned to five groups, with three ducklings in each group, for safety evaluation over a 28-day period. Four experimental groups were immunized with 100 µg of OMVs: one group received OMVs without antigen proteins (Lpp-OMVs), while the other three groups were immunized with OMVs displaying one of the following recombinant antigens: YaeT (Lpp-YaeT), PorQ (Lpp-PorQ), or YiaD (Lpp-YiaD). The fifth group served as a PBS control and received an equal volume of sterile PBS. Body weight was monitored every 7 days for 28 days following immunization to assess potential effects on growth and overall health.

Following the safety evaluation, the ducklings were divided into five groups, with 20 ducklings per group. Four groups were intramuscularly immunized with 100 μg of one of the following: OMVs-YaeT, OMVs-PorQ, OMVs-YiaD, or OMVs from the engineered *Salmonella* strains without antigens (vector control). One group received sterile PBS and served as the challenge control. Another group served as the blank control and received PBS without subsequent challenge. A booster immunization with the same formulation was administered on day 14. Fourteen days after the booster immunization, all groups except the blank control were challenged with the virulent RA serotype 2 strain RA-SD-1 (5 × LD_50_) to assess protective efficacy.

### Determination of serum antibody levels

To evaluate the antigen-specific humoral immune response, blood samples were collected from the wing vein of each duckling at 7 days after primary immunization and 7 days after booster immunization. Serum was separated by centrifugation at 3,000 × *g* for 10 min and stored at −20°C until analysis. Antibody levels against YaeT, PorQ, and YiaD were determined by indirect enzyme-linked immunosorbent assay (ELISA). Briefly, 96-well microtiter plates were coated overnight at 4°C with 100 μL per well of the corresponding recombinant protein (2 μg/mL) diluted in carbonate-bicarbonate buffer (pH 9.6). After blocking with 5% skim milk in PBST at 37°C, serum samples diluted at a fixed ratio were added and incubated at 37°C for 1 h. After washing with PBST, the plates were incubated with horseradish peroxidase (HRP)-conjugated goat anti-duck IgY (Abcam, UK) for 1 h at 37°C. The reaction was developed with TMB substrate and stopped with 2 M H_2_SO_4_. The absorbance was measured at 450 nm using a microplate reader, and OD_450_ values were used to represent antigen-specific antibody levels.

### Lymphocyte proliferation assay

Peripheral blood mononuclear cells (PBMCs) were isolated from ducklings on day 21 (7 days post-booster immunization) using a PBMC isolation kit (Solarbio, Beijing, China). Blood was collected from the wing vein, and isolation was performed according to the manufacturer’s instructions (56). The PBMC layer was collected, washed twice with PBS, and resuspended in RPMI-1640 medium (Gibco) supplemented with 10% fetal bovine serum, penicillin (100 U/mL), and streptomycin (100 μg/mL) at a final concentration of 1 ×  10^7^ cells/mL.

For the proliferation assay, 1 ×  10^5^ PBMCs per well were seeded in 96-well plates and stimulated in triplicate with 0.5 μg/well of the corresponding recombinant protein (YaeT, PorQ, or YiaD) at 37 °C in a 5% CO_2_ incubator for 72 h. RPMI-1640 medium alone was used as the negative control, and 2 μg/mL concanavalin A (ConA; Sigma-Aldrich, USA) was used as the positive control. After incubation, 10 μL of Cell Counting Kit-8 (CCK-8; Sangon Biotech Co., Ltd., Shanghai, China) solution was added to each well, and the plates were incubated for an additional 2 h. The absorbance was measured at 450 nm using a microplate reader. The stimulation index (SI) was calculated as follows: SI = (OD of treatment − OD of blank)/(OD of treatment − OD of treatment).

### Cytokine assay

To assess cytokine responses at the transcriptional level, PBMCs were collected 7 days after booster immunization and stimulated *in vitro* with 1 μg/well of the corresponding recombinant protein at 37 °C with 5% CO_2_ for 3 h. The mRNA expression levels of IFN-γ, TNF-α, IL-4, and IL-6 were determined by quantitative real-time PCR (qRT-PCR) as previously described with minor modifications. Total RNA was extracted using RNA-easy Isolation Reagent (Vazyme, China) according to the manufacturer’s protocol, and cDNA was synthesized using the PrimeScript RT reagent kit (Takara, Japan). qRT-PCR was performed using SYBR Green PCR Master Mix (Vazyme, China) and gene-specific primers listed in Table 2. GAPDH was used as the internal reference gene.

### Bacterial load determination

Five surviving ducklings were randomly selected on days 2 and 4 post-infection and euthanized by CO_2_ inhalation. The heart and liver were collected and placed in sterile 50 mL centrifuge tubes. After weighing, PBS was added at a ratio of 0.9 mL per 0.1 g of tissue. The samples were then transferred to sterile tubes and homogenized using the MP FastPrep-24 (MP Biomedicals, Santa Ana, CA, USA). The tissue samples were serially diluted in PBS and plated onto TSA agar plates containing 5% fetal bovine serum and 50 μg/mL kanamycin, as RA is naturally resistant to kanamycin (57). The plates were incubated overnight at 37°C to determine bacterial CFU counts.

### Survival rate and histopathological examination

Duckling survival was assessed every 12 h over 14 days following challenge with RA-SD-1, which is a Serotype 2 strain of RA. The number of surviving ducklings in each group was recorded, and the cumulative survival rate was calculated as the percentage of survivors relative to the total number of ducklings in each group.

Upon death, ducklings were immediately necropsied to assess gross pathological changes. Tissues from the liver and heart were collected, fixed in 10% formalin, and embedded in paraffin. Sections (5 μm) were prepared and stained with hematoxylin and eosin (H&E). Histopathological lesions were examined under a light microscope at ×400 magnification, and tissue damage was scored as follows: 0 = no lesion, 1 = lesion area <33%, 2 = lesion area 33–66%, and 3 = lesion area >66%.

### Statistical analysis

All data were expressed as mean ± standard deviation (SD). Statistical analyses were performed with the statistical software program Prism, version 5.0 (GraphPad Software Inc., San Diego, CA, USA). One-way analysis of variance (ANOVA) followed by Tukey’s multiple-comparison test was used to evaluate differences among groups. A *P* value of less than or equal to 0.05 (*) was considered a significant difference, and a *P* value of less than or equal to 0.01 (**) represents a highly significant difference. *P* values larger than 0.05 were considered to be not significant (n.s).

## Data Availability

All data generated or analyzed during this study are available from the corresponding author by request.

## SUPPLEMENTAL MATERIAL

Fig. S1 aem.00608-26-s0001.docx Lymphocyte proliferation in PBS and Lpp-OMVs control groups.

ASM does not own the copyrights to Supplemental Material that may be linked to, or accessed through, an article. The authors have granted ASM a non-exclusive, world-wide license to publish the Supplemental Material files. Please contact the corresponding author directly for reuse.
