# Supplementary material for: Salmonella-derived OMVs as a vaccine platform for Riemerella anatipestifer outer membrane proteins to prevent infection in ducks
Source: Appl Environ Microbiol. 2026 Jun 24;92(7):e00608-26. doi: 10.1128/aem.00608-26 (PMC13390429; doi:10.1128/aem.00608-26)
Supplement: Fig. S1 — Lymphocyte proliferation in PBS and Lpp-OMVs control groups. [file aem.00608-26-s0001.docx]

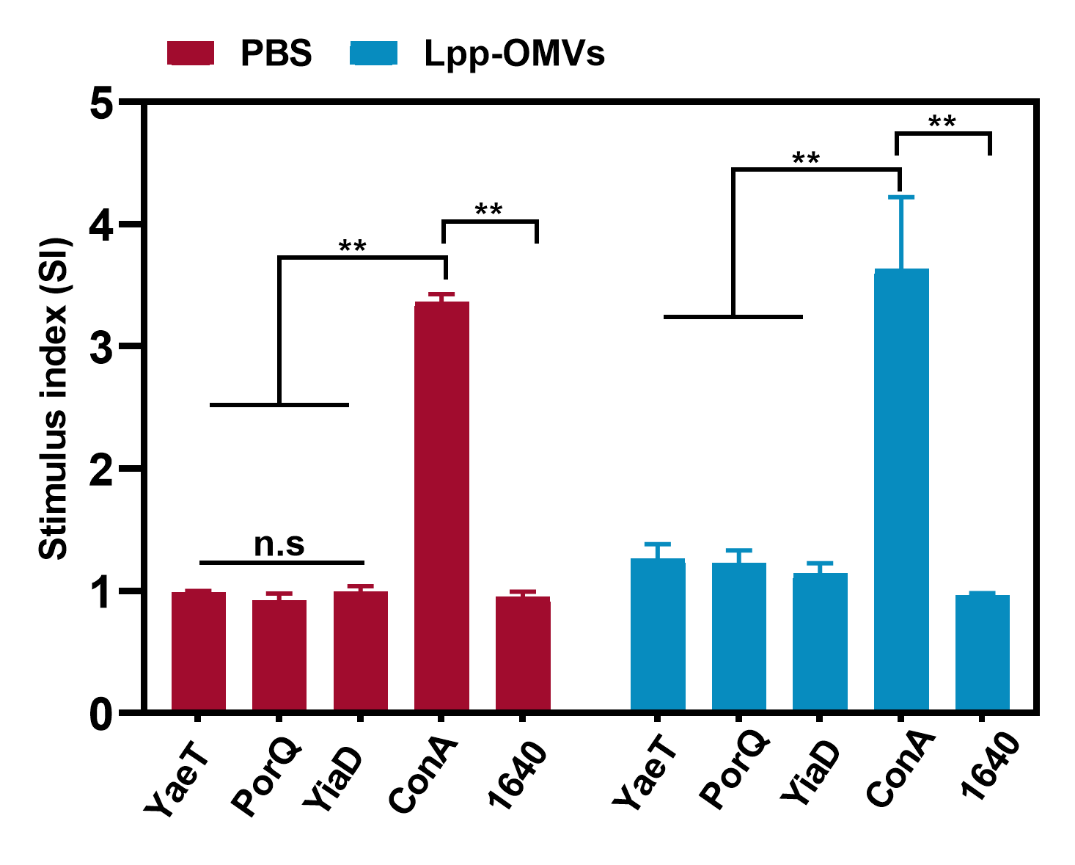


**Figure S1. Lymphocyte proliferation in PBS and Lpp-OMVs control groups.**

PBMCs were isolated from ducks in the PBS and Lpp-OMVs groups and stimulated *in vitro* for 24 h with the corresponding recombinant antigens (YaeT, PorQ, or YiaD), ConA, or 1640. Lymphocyte proliferation was assessed using a CCK-8 assay, and SI were calculated as for the antigen-carrying OMV groups.
